# Anabolic Androgenic Steroids Induce Reversible Left Ventricular Hypertrophy and Cardiac Dysfunction. Echocardiography Results of the HAARLEM Study

**DOI:** 10.3389/frph.2021.732318

**Published:** 2021-09-01

**Authors:** Diederik L. Smit, A. J. Voogel, Martin den Heijer, Willem de Ronde

**Affiliations:** ^1^Department of Internal Medicine, Spaarne Gasthuis, Haarlem, Netherlands; ^2^Department of Cardiology, Spaarne Gasthuis, Haarlem, Netherlands; ^3^Department of Internal Medicine, Amsterdam University Medical Centres, Amsterdam, Netherlands

**Keywords:** anabolic steroids, performance and image-enhancing drugs, strength athletes, bodybuilding, three-dimensional echocardiography

## Abstract

**Background:** The use of anabolic androgenic steroids (AAS) is not uncommon among strength athletes. Several cross-sectional studies have linked AAS use to heart disease, but a causal role for AAS is not certain and it is unknown whether cardiac changes are reversible.

**Methods:** Men of at least 18 years old intending to start an AAS cycle on short notice were included for comprehensive 3D echocardiographic examination before (T_0_), at the end of the cycle (T_1_), and 1 year after inclusion (T_2_) after a recovery period. Details of the AAS cycle performed and the use of other performance and image-enhancing drugs (PIEDs) as well as illicit drug use were recorded. Trend analysis and multivariable regression analysis were performed with mixed effects linear models.

**Results:** Thirty-one subjects were included. Between start (T_0_) and end of the cycle (T_1_), after a median AAS cycle duration of 16 weeks, 3D left ventricular ejection fraction declined with 4.9% (CI −7.2 to −2.5, *P* < 0.001), E/A-ratio declined with−0.45 (CI −0.69 to −0.21, *P* < 0.001), and 3D left atrial volume increased with 9.2 ml (CI 2.9–15.4, *P* = 0.004). Left ventricular mass increased with 28.3 g (CI 14.2–42.4, *P* < 0.001) and was positively correlated with AAS average weekly dose. After a median recovery time of 8 months (T_2_), all parameters returned to baseline.

**Conclusion:** AAS induce left ventricular hypertrophy and impaired systolic and diastolic function in amateur strength athletes. The structural cardiac changes are positively associated with AAS dose and complete recovery occurred after AAS were discontinued.

## Introduction

The use of anabolic androgenic steroids (AAS) is not uncommon among strength athletes and regular visitors of fitness centers. The lifetime prevalence for men is estimated at 3% (1). Production and trading of AAS without a license is prohibited in most countries, but AAS can be illegally acquired through local dealers or the internet.

It is beyond doubt that AAS are harmful. Current knowledge however is based on rather low levels of evidence including expert opinion, case reports and case control studies. Prospective studies describing adverse effects of AAS are scarce. The use of AAS is associated with agitation, mood and anxiety disorders (2), liver toxicity and hepatocellular neoplasia (3, 4), and disruption of gonadal function after cessation of use (5, 6). AAS use could also pose a risk factor for cardiovascular disease due to unfavorable effects on blood pressure, haematocrit and lipid metabolism (7). A cross-sectional case control study using computed tomography angiography showed higher coronary artery plaque volumes in weightlifters using AAS compared to non-users (8).

Several reports have also linked AAS use to heart disease, such as left atrial dysfunction (9), ventricular diastolic (10) and systolic dysfunction (11–13), impaired ventricular strain (14) and left ventricular hypertrophy (15, 16). These studies are all cross-sectional in nature and a causal role for AAS is thus not certain. It is also not known whether cardiac changes are reversible. An additional limitation is the heterogeneous and often poorly documented records on AAS use of the strength athletes in these study groups.

The HAARLEM study is a prospective observational cohort study that started in 2015 and investigated the effects of AAS use by amateur strength athletes (4). We recruited male athletes who intended to start an AAS cycle on short notice. Baseline measurements were performed prior to the start of the AAS cycle. Repeat analyses were performed in the last week of the cycle and 1 year after the start of the cycle. The study posed a unique opportunity to obtain prospective data about both structural and functional cardiac changes during AAS use and therefore we performed comprehensive echocardiographic examinations at these three points in time.

All androgens used by subjects in our study were illegal and not registered for use in humans. It would therefore be unethical to conduct a controlled intervention study in which subjects would use androgens according to a study protocol. Prospective, observational studies like the HAARLEM study may thus be the only feasible approach to collect valid data (17).

## Methods

Details of subject recruitment of the HAARLEM study are described in a previous report (4). In short, men of at least 18 years old intending to start an AAS cycle on short notice (i.e., within 2 weeks) were invited to sign up after the study was promoted on national television, regional newspapers and social media. We did not interfere with the chosen dose, duration or contents of the planned AAS cycle. However, to guarantee significant exposure, the cycle had to be at least 6 weeks in duration, average a weekly dose of a minimum of 200 mg of androgens, and comprise at least two different types of AAS. Subjects were excluded if they had used AAS in the 3 months before inclusion or had suffered any major somatic or psychological health issues in the previous 6 months.

The HAARLEM study started in October 2015 and 100 men were included for health analysis. Echocardiography was added to the analysis in May 2017 after approval by the local Medical Ethics Committee. From this point until April 2018, the last 31 subjects included in the HAARLEM study were offered analysis with echocardiography, all of whom provided informed consent.

### Clinic Visits

Subjects underwent 3 echocardiographic examinations during a 1 year study period. The baseline visit took place immediately after inclusion and before the initiation of the AAS cycle (T_0_). Subjects returned to the clinic in the last week of the AAS cycle (T_1_). The cycle performed was recorded in detail, with duration and dosage of each type of AAS used, in addition to use of other performance and image enhancing drugs (PIEDs), medication and post-cycle therapy (PCT). Symptoms or side effects experienced by subjects were recorded. After clinic visit T_1_ subjects discontinued AAS use for the remaining part of the study period. The last visit followed 1 year after the start of the cycle (T_2_). As a result, the interval between T_1_ and T_2_ was variable, i.e., the recovery phase, dependent on the duration of the AAS cycle.

### Echocardiography

Cardiac 3D echocardiography was performed on a state-of-the-art Philips Epiq 7 device. The HeartModel application was used for cardiac chamber 3D quantification. Two EACVI certified analysts carried out the echocardiography and echocardiograms of the same subject were made by the same analyst as much as possible to reduce interobserver variability. Interpretation of the echocardiographic data was performed by the study cardiologist after full data collection and after blinding for the type of clinic visit.

Resting heart rate (/min), blood pressure (mmHg), height (cm), weight (kg) and body surface area (BSA, m^2^) were obtained during each clinic visit. The following variables were extracted from the obtained echocardiogram (with corresponding units):

- Left ventricle:

◦ Left ventricular end-diastolic dimension (LVEDd, mm), left ventricular end-systolic dimension (LVEDs, mm), left ventricular end-diastolic volume 3D (LVEDV 3D, ml), left ventricular end-systolic volume 3D (LVESV 3D, ml), intraventricular end-diastolic septal thickness (IVSd, mm), left ventricular end-diastolic posterior wall thickness (LVPWD, mm), left ventricular mass (LV mass, g), left ventricular ejection fraction 3D (3D LVEF, %), global longitudinal strain (LV strain global, %), myocardial performance index (MPI, Tei-index, no dimension).

- Diastolic function:

◦ Mitral valve E wave (E, cm/s), mitral valve E duration time (E-DT, ms), mitral valve A wave (A, cm/s), mitral valve A wave duration time (A-DT, ms), E/A-ratio, lateral e' wave (e' lat, cm/s), septal e' wave (e' sept, cm/s), lateral E/e'-ratio (E/e' lat), septal E/e'-ratio (E/e' sept), pulmonary vein Arev (PV Arev, cm/s), pulmonary vein Arev duration time (PV Arev DT, ms), pulmonary vein D (PVD, cm/s), pulmonary vein S (PVS, cm/s), left atrial volume index (LAVI, ml/m^2^), left atrial volume 3D (LAvol3D, ml), LAD parasternal long-axis view (LAD PSLAX).

- Right ventricle:

◦ Right ventricular tricuspid annular plane systolic excursion (RV TAPSE, mm), right ventricular tissue Doppler imaging (RV TDI, cm/s), right ventricular annulus diastolic (RV annulus, mm), right ventricular fractional area change (RVFAC, %), pulmonary artery acceleration slope (PA acc slope, cm/s^2^), pulmonary artery acceleration time (PAAT, ms), tricuspid valve insufficiency severity (TV severity, 0–4), systolic pulmonary artery pressure (SPAP), right atrium pressure (RA pressure, mmHg), right ventricular outflow tract (RVOT, mm).

Mitral inflow velocities were recorded in the apical 4-chamber view with the pulsed-wave Doppler sample volume placed at the level of the mitral valve tips. The peak velocity of early (E) and late (A) diastolic waves and the E-DT were measured. The peak myocardial systolic (s′), early diastolic (e′), and late diastolic (a′) velocities were measured in the apical 4- and 2-chamber views using Doppler tissue imaging (DTI). LV mass was calculated using the Devereux cube formula incorporated in the IntelliSpace workstation.

### Analysis

Simple descriptive statistics were used to display quantitative data. If the variables were normally distributed, mean and standard deviation were calculated. If the distribution of a variable was skewed, a median is presented with range. For the comparison of the results of clinic visits, mixed effects linear models were used to calculate 95%-confidence intervals (CI) and *P*-values. In this model missing data are accounted for with maximum likelihood estimation.

Multivariable regression analysis was also performed with mixed models, assessing the effects of AAS cycle dose and length, number of AAS used, whether or not oral AAS were used, previous AAS use, mean arterial blood pressure (MAP), cumulative history of AAS use, training time, concurrent use of growth hormone (GH), ecstasy (XTC), cocaine, and the use of post-cycle therapy (PCT). In the multivariable regression analysis, to correct for multiple testing, only a *P*-value < 0.01 was considered statistically significant.

## Results

Thirty-one subjects of the HAARLEM study were included for echocardiography. Relevant baseline characteristics and AAS cycle characteristics are shown in Table 1. Subjects were on average 33 years old. All of them were active in strength sports, averaging 307 min per week at the gym at baseline, which increased to 390 min per week during the cycle (*P* = 0.002) and decreased to 243 min after the cycle (*P* = 0.010). Twenty-seven subjects had performed an AAS cycle before. The cycle performed during the study period had a median duration of 16 weeks and median weekly dose of 904 mg testosterone equivalents. This was similar to the cycle characteristics of the subjects in the HAARLEM study cohort who were not included for echocardiography. In this group the median cycle duration was 15 weeks and the median weekly dose was 898 mg (unpaired *t*-test, *P* = 0.31 and *P* = 0.81, respectively).

**Table 1:** Baseline characteristics, AAS cycle characteristics and details of drug abuse of the 31 subjects of the HAARLEM study included for echocardiography.

**General**	***n* (%), mean (SD, ranges)**
Male	31 (100%)
Age	33 (±8.4; 20–67)
Height (cm)	182 (±6.8; 171–193)
Weight (kg)	88.2 (±10.5; 71–118)
Body mass index (BMI, kg/m^2^)	26.6 (±2.2; 23.1–32.7)
Previous AAS use	27 (87%)
**Current sport**	***n*** **(%)**
Fitness/bodybuilding	31 (100%)
Competitive bodybuilding	3 (10%)
Weight lifting	1 (3%)
Combat sports (e.g., kickboxing, karate, judo)	12 (39%)
**Fitness schedule at baseline**	**Mean (SD, ranges)**
Number of training sessions (/week)	4 (±1.1; 1–7)
Duration of training sessions (minutes)	73 (±15; 60–105)
Time weekly spent at gym (minutes)	307 (±110; 80–630)
**Cycle characteristics**	***n*** **(%), median (ranges)**
Cycle length (weeks)	16 (±8.0, 7–42)
Number of AAS	4 (1–11)
Average weekly dose (mg)*	904 (250–3,382)
Cumulative dose (mg)*	13.200 (3.000–74,410)
Post-cycle therapy	26 (84%)
**Use of other PIEDs during cycle**	***n*** **(%)**
Creatine	9 (29%)
Growth hormone	8 (26%)
Levothyroxine	4 (13%)
hCG	3 (10%)
Insulin	2 (6%)
Tamoxifen	6 (19%)
Aromatase inhibitors (anastrozole, exemestane)	9 (29%)
**Drug use during study period**
Nicotine	13 (42%)
Alcohol	21 (68%)
Ecstasy/amphetamines	13 (42%)
Cocaine	11 (35%)
Gamma-hydroxybutyric acid (GHB)	11 (35%)
Cannabis	10 (32%)
Other (3-FMP, ketamine, 2-CB)	7 (23%)
≥3 drugs (except nicotine and alcohol)	8 (26%)

* *Cumulative dose is the sum of all different AAS compounds used in a cycle in mg. For practical purposes, all types of AAS were regarded as having a 1 to 1 equivalence with testosterone (e.g., 1 mg testosterone = 1 mg nandrolone = 1 mg stanozolol). The average weekly dose is calculated by dividing the cumulative dose by cycle length in weeks*.

Analysis with echocardiography was performed in all 31 subjects at the start (T_0_) and the end of the cycle (T_1_). The median time between T_1_ and T_2_, corresponding to recovery time after the AAS cycle, was 8 months (range 5–12). Four subjects started a new cycle before T_2_, hence echocardiography was not carried out in them as it could not assess recovery. Another 2 subjects missed T_2_ due to obligations for work and emigration, respectively. Consequently, 25 subjects were analyzed with echocardiography after the recovery period (T_2_). As intended, T_2_ took place after a median of 12 months (range 8–16) after inclusion (T_0_), i.e., total follow-up period.

Details on the side effects reported by subjects of the HAARLEM study are published separately (18) but none reported dyspnoea or peripheral oedema at the end of the cycle (T_1_). Two of 31 subjects complained of fatigue. Fluid retention was reported by 15 of 31 subjects. Between the start (T_0_) and the end (T_1_) of the cycle, there was an increase in heart rate of 10.0 beats/min (CI 5.8–14.2, *P* < 0.001) and BSA of 0.05 m^2^ (CI 0.01–0.10, *P* = 0.020). BSA at the end of the cycle (T_1_) was positively associated with AAS cycle dose and GH use, but negatively associated with training time. Mean systolic and diastolic blood pressure increased with 6 mmHg (CI 1.2–11.0, *P* = 0.015) and 5 mmHg (CI 0.9–8.2, *P* = 0.013), respectively. Heart rate, blood pressure and BSA returned to baseline after the recovery period (T_2_).

Detailed results of the trend analysis and multivariable regression analysis of all measured echocardiographic parameters are shown in Supplementary Tables 1, 2, respectively. Important findings are elaborated in the subsequent paragraphs.

### Left Ventricle

**Figure 1:**
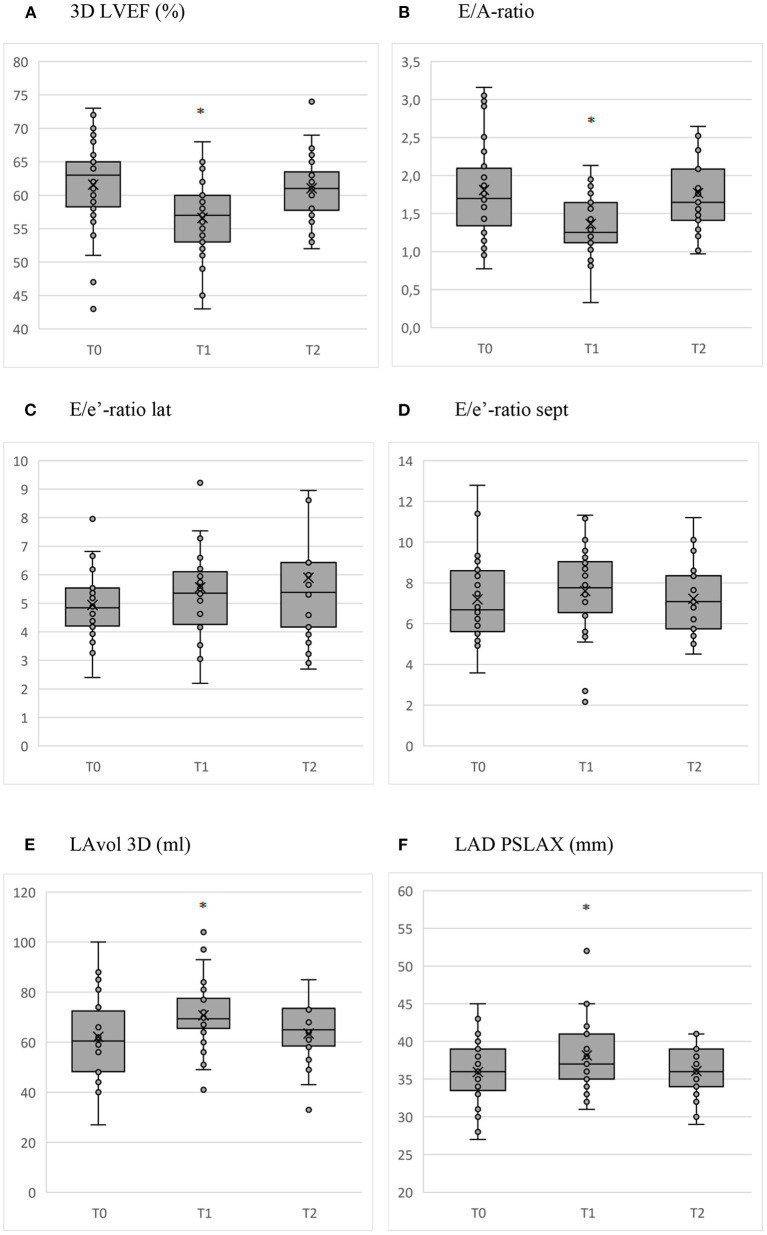
Box and whisker plots of the relevant parameters measured with echocardiography before (T_0_) and at the end of the cycle (T_1_) with AAS, and after the recovery period (T_2_). *P < 0.01. **(A)** 3D LVEF (%). **(B)** E/A-ratio. **(C)** E/e'-ratio lat. **(D)** E/e'-ratio sept. **(E)** LAvol 3D (ml). **(F)** LAD PSLAX (mm).

The 3D LVEF trend is shown in Figure 1A. 3D LVEF declined with 4.9% (CI −7.2 to −2.5, *P* < 0.001). 3D LVEF was negatively associated with training time at the end of the cycle (T_1_).

Between the start (T_0_) and end of the cycle (T_1_), there was an increase in 3D LVEDV and 3D LVESV of 10.3 ml (CI 1.0–19.7, *P* = 0.030) and 11.5 ml (CI 6.8–16.2, *P* < 0.001), respectively. After the recovery period (T_2_), these volumes declined with −16.9 ml (CI −26.5 to −7.2, *P* = 0.001) and −8.0 ml (CI −12.9 to −3.0, *P* = 0.002) compared to T_0_, respectively. GH use was associated with a higher 3D LVEDV and 3D LVESV at the end of the cycle (T_1_), whereas the number of AAS used in the cycle was negatively associated with 3D LVEDV and 3D LVESV both measured at the end of the cycle (T_1_) as after the recovery period (T_2_).

LV mass increased with 28.3 g (CI 14.2–42.4, *P* < 0.001) between the start (T_0_) and the end of the cycle (T_1_). This trend was not different when LV mass was adjusted for BSA, i.e., g/m^2^. IVSd and LVPWD both increased during the cycle (T_1_) with 0.87 mm (CI 0.44–1.30, *P* < 0.001) and 1.18 mm (CI 0.76–1.61, *P* < 0.001), respectively. There was a positive correlation between AAS average weekly dose and LV mass, LVPWD and IVSd at the end of the cycle (T_1_). Contrarily, IVSd was negatively associated with training time at the end of the cycle (T_1_) and after the recovery period (T_2_). XTC use also had a negative impact on LV mass measured at the end of the cycle (T_1_). LV mass, IVSd and LVPWD all returned to baseline after the recovery period (T_2_).

The parameters LVEDd, LVEDs, LV strain global and MPI did not show a significant change during the course of clinic visits.

### Diastolic Function

The trend for E/A-ratio during clinic visits is shown in Figure 1B. A increased with 9.1 cm/s (CI 2.6–15.7, *P* = 0.006) during the cycle (T_1_). No significant changes occurred in A-DT, E and E-DT. The resulting decline of the E/A-ratio between the start (T_0_) and the end of the cycle (T_1_) was −0.45 (CI −0.69 to −0.21, *P* < 0.001) with normalization to baseline after the recovery period (T_2_).

The changes of the E/e' lat and sept through the course of clinic visits are shown in Figures 1C, D. None of these changes were statistically significant. There was a decline of e' lat during the cycle (T_1_) with −1.8 cm/s (CI −3.5 to −0.1, *P* = 0.038). Age, cocaine use and training time were positively associated with E/e' sept at the end of the cycle (T_1_), whereas AAS average weekly dose was negatively associated with E/e' sept.

The change of LAvol3D and LAD PSLAX during clinic visits is shown in Figures 1E, F. During the cycle (T_1_), LADvol3D increased with 9.2 ml (CI 2.9–15.4, *P* = 0.004) and LAD PSLAX with 1.9 mm (CI 0.7–3.2, *P* = 0.002). The increase in LAD PSLAX was greater for subjects using oral AAS (β = 3.7, CI, 1.2–6.2, *P* = 0.004). The parameters normalized after the recovery period (T_2_).

The parameters PV Arev, PV Arev DT, PVD, and PVS did not show any significant change during the study period.

### Right Ventricle

During the course of clinic visits, there were no significant changes in RV TAPSE, TV TDI, RV annulus, RVFAC, PA acc slope, PAAT, RVOT and RVAC. Tricuspid valve insufficiency was grade 1 in only 4, 2, and 4 subjects at the start (T_0_) and end of the cycle (T_1_), and after the recovery period (T_2_), respectively, so SPAP and RA pressure could not be analyzed reliably.

## Discussion

The main findings of this study were that an AAS cycle with supraphysiological doses of androgens induced an increase in left ventricular mass, a reduction of left ventricular ejection fraction by ~5%, and a higher left ventricular stiffness reflected by the reduction of E/A-ratio, although changes in filling pressures cannot be excluded. There was a positive relationship between average weekly dose of AAS and left ventricular mass. When AAS were discontinued, after a median recovery time of 8 months, all parameters returned to their baseline values.

The increase in left ventricular mass was due to an increase in interventricular septum as well as posterior wall thickness. This is in compliance with the results of a recent large cross-sectional study by Baggish et al. (8). This group also showed a reduction of left ventricular ejection fraction in AAS users. A lower E/A-ratio has also been documented in previous studies comparing bodybuilders to non-users and sedentary controls (10, 19).

The data from our cohort study are novel because the prospective single-subject design allowed to assess not only the effect of AAS on cardiac function but also whether recovery occurred after the cycle. This showed complete normalization of all parameters after discontinuation of AAS. In addition, we meticulously characterized the AAS cycles performed by the subjects and as such we could demonstrate that androgen dose was associated with increased left ventricular mass and diameters.

### Limitations

Several shortcomings of the HAARLEM study are mentioned in the baseline data report (4). The main limitation was the fact that the study design precluded the possibility to establish causality between androgen abuse and the observed cardiac effects. The course of the data in time, however, as well as the association of left ventricular parameters and androgen dose, leave little doubt that androgen abuse was the main determinant of the observed cardiac changes. Moreover, androgens were shown to independently cause cardiac hypertrophy and impaired systolic and diastolic function in rat studies (20).

Furthermore, several other variables play a confounding role in cardiac function, such as blood pressure and exercise. Although mean blood pressure increased mildly during AAS use, we did not find it to interact significantly with cardiac function. This was probably because rise in blood pressure was fairly small and existed for a relatively short amount of time. Long-term intensive exercise may also induce myocardial changes of the left ventricle, i.e., the Morganroth hypothesis. Even though our subjects spent an average 7.5 h in the gym per week during the cycle, it is unlikely that the observed cardiac changes were a result of vigorous strength training. First, there is evidence indicating that the effect of exercise on the heart particularly applies to endurance athletes and not strength athletes (21). Secondly, included subjects usually had a long history of strength training and the actual increase in training time during the cycle was only modest in comparison to their training time before the cycle. Cardiac changes reversed after discontinuation of AAS even though subjects kept an intensive training regimen. Thirdly, our multivariable analysis did not show an association between training time and observed myocardial changes.

### Clinical Relevance

Our findings strongly support the cardiotoxic nature of AAS. The changes in cardiac structure and function did not lead to symptoms, however, such as dyspnoea or peripheral oedema, as these were not reported by the subjects. The occurrence of heart failure in users of AAS nevertheless has often been reported (22). We hypothesize that cumulative cardiac damage may follow long-standing AAS use when recovery time in between cycles is too short or when AAS are used continuously. Our data could not substantiate the presence of such cumulative damage as we did not observe a relationship between the extent of prior AAS use and cardiac abnormalities at baseline. Of note, our cohort displayed a wide variety of historic AAS use, ranging from no prior use to 8 years of cumulative AAS use, but no subjects had abused androgens continuously for longer than 1 year. It is therefore likely that progression to clinical heart failure only occurs in those athletes with an excessive history of AAS use, or when an athlete has a prior medical condition affecting the heart, e.g., cardiomyopathy.

## Conclusion

We have shown that AAS induce left ventricular hypertrophy and impaired systolic and diastolic function in amateur strength athletes. This effect was independent from changes in blood pressure and training time. The structural cardiac changes were greater if larger androgen doses were used. Impaired cardiac function did not lead to clinical signs of heart failure. All parameters normalized within the 1 year follow-up period after the AAS were discontinued.

## Data Availability Statement

The raw data supporting the conclusions of this article will be made available by the authors, without undue reservation.

## Ethics Statement

The studies involving human participants were reviewed and approved by METC Noord-Holland (registration number M015-019). The patients/participants provided their written informed consent to participate in this study.

## Author Contributions

DS, WR, and AV contributed to the conception and design of the study. AV collected and reviewed the data. DS organized the database, performed the statistical analysis, and wrote the first draft of the manuscript. WR wrote sections of the manuscript as well. All authors contributed to manuscript revision, read, and approved the submitted version.

## Conflict of Interest

The authors declare that the research was conducted in the absence of any commercial or financial relationships that could be construed as a potential conflict of interest.

## Publisher's Note

All claims expressed in this article are solely those of the authors and do not necessarily represent those of their affiliated organizations, or those of the publisher, the editors and the reviewers. Any product that may be evaluated in this article, or claim that may be made by its manufacturer, is not guaranteed or endorsed by the publisher.
