# Supplementary material for: Anabolic Androgenic Steroids Induce Reversible Left Ventricular Hypertrophy and Cardiac Dysfunction. Echocardiography Results of the HAARLEM Study
Source: Front Reprod Health. 2021 Sep 1;3:732318. doi: 10.3389/frph.2021.732318 (PMC9580689; doi:10.3389/frph.2021.732318)
Supplement: Supplementary file 2 [file Table_2.DOCX]

*Supplementary table 2.* Results of multivariable regression analysis. Variables included in the analysis were age, cumulative history of AAS use, mean arterial blood pressure (MAP), cycle length, average weekly dose, number of AAS used, use of oral AAS at time of echocardiography, use of post-cycle therapy (PCT), training time, and cocaine, XTC or growth hormone (GH) use during the study period. All parameters shown in supplementary table 1 were analysed for confounding. This table shows the variables and respective clinic visit for which there was a statistically significant interaction (P<.01 to correct for multiple testing).

| **Variables** | **Clinic visit** | **Parameter** | **Coefficient [CI]** |
| --- | --- | --- | --- |
|  | | | |
| Age | T0 | PVS (cm/s) 3D LAvol | β = 0.59 [0.23 to 0.96] β = 0.86 [0.36 to 1.37] |
|  | T1 | E/e’ sept | β = 0.21 [0.09 to 0.34] |
|  | | | |
| Weekly AAS dose | T1 | BSA (m^2^)  IVSd (mm) LVPWD (mm)  LV mass (g) E (cm/2) E/e’ sept | β = 7.8e^-5^ [2.2e^-5^ to 1.3e^-4^]  β = 1.4e^-3^ [9.4e^-4^ to 1.8e^-3^]  β = 1.0e^-4^ [3.8e^-4^ to 1.6e^-3^] β = 3.0e^-2^ [1.0e^-2^ to 4.9e^-2^]  β = -2.0e^-2^ [-3.0e^-2^ to -9.7e^-3^] β = -2.4e^-3^ [-4.0e^-3^ to -7.4e^-4^] |
|  | | | |
| Number of AAS | T1 | 3D LVEDV (ml) 3D LVESV (ml) | β = -13.1 [-18.8 to -7.45] β = -7.24 [-9.57 to -4.91] |
|  | T2 | 3D LVEDV (ml)  3D LVESV (ml) | β = -10.7 [-16.3 to -5.11]  β = -3.41 [-5.70 to -1.13] |
|  | | | |
| Length of cycle | T1 | 3D LVESV (ml) | β = 1.47 [1.00 to 1.93] |
|  | | | |
|  | | | |
| Training time | T1 | BSA (m^2^)  IVSd (mm)  E (cm/s) E/e’ sept  3D LVEF (%) | β = -5.1e^-4^ [-8.5e^-4^ to 1.8e^-4^]  β = -3.9e^-3^ [-6.4e^-3^ to 1.4e^-3^] β = 9.4e^-2^ [3.7e^-2^ to 0.15] β = 1.4e^-2^ [5.8e^-3^ to 2.2e^-2^] β = -3.1e^-2^ [-4.8e^-2^ to -1.4e^-2^] |
|  | T2 | IVSd (mm) | β = -5.1e^-3^ [-7.5e^-3^ to -2.8e^-3^] |
|  | | | |
| GH use | T1 | BSA (m^2^) LVEDd (mm)  3D LVEDV (ml) 3D LVESV (ml) | β = 0.19 [0.08 to 0.30] β = 7.99 [2.33 to 13.7]  β = 38.6 [15.1 to 62.1]  β = 24.2 [14.1 to 34.4] |
|  | T2 | 3D LVESV (ml) | β = 20.1 [9.81 to 30.5] |
|  | | | |
| Cocaine use | T1 | E (cm/s) E/e’ sept | β = 21.7 [7.68 to 33.7] β = 2.97 [1.00 to 4.94] |
|  | T2 | E (cm/s) | β = 22.9 [7.36 to 38.4] |
|  | | | |
| XTC use | T1 | LVEDd (mm) 3D LVESV (ml)  LV mass (g) | β = -7.37 [-11.7 to -3.02]  β = -11.5 [-20.2 to -2.82]  β = -51.1 [-80.0 to -22.1] |
